# Phaeoacremonium species detected in fine needle aspiration: a rare case report

**DOI:** 10.1186/s13000-020-01023-w

**Published:** 2020-09-20

**Authors:** Santosh Tummidi, Bitan Naik, Arundhathi Shankaralingappa, Pavithra Balakrishna, Arati Ankushrao Bhadada, Navya Kosaraju

**Affiliations:** 1 Department of Pathology, All India Institute of Medical Sciences, Mangalagiri, Andhra Pradesh 522503 India; 2 Department of General Surgery, All India Institute of Medical Sciences, Mangalagiri, Andhra Pradesh 522503 India; 3 Department of Microbiology, All India Institute of Medical Sciences, Mangalagiri, Andhra Pradesh 522503 India; 4 Department of Radiology, All India Institute of Medical Sciences, Mangalagiri, Andhra Pradesh 522503 India

**Keywords:** Phaeoacremonium, Fungal, Toluidine blue, Fine needle aspiration, Rapid-on-site

## Abstract

**Background:**

Fine needle aspiration cytology (FNAC) with rapid on-site evaluation has a great potential for the diagnosis of fungal lesions and other opportunistic infections. Fungal infections have been in increasing trend in the past two decades due to immunosuppression, travel, and environmental exposure. Human disease caused by Phaeoacremonium species is rare and was first reported in 1974 as subcutaneous tissue infection in a renal transplant recipient.

**Case presentation:**

We report a case of subcutaneous tissue swelling in a 67-year-old male, wherein FNAC was done with incidental detection of the fungus (Phaeoacremonium spp).

**Conclusion:**

There are very few reported cases of subcutaneous infection in humans by Phaeoacremonium spp. Clinical suspicion and FNAC can play an important role in early detection of the fungus, prevent spread, and facilitating early treatment.

## Background

Phaeohyphomycosis is a rare form of sporadic infection that is caused by dematiaceous fungi. It’s commonly detected in tropics and sub-tropical environments. It’s a saprophyte in soil and vegetation causing Petri or esca diseases in plants [1, 2]. The first reported case was in 1974 by Ajello et al in a renal transplant patient [3]. Most often, the diagnosis is delayed due to the rarity and variable nature of the presentation [4, 5].

FNAC can play a definitive role in early identification of the disease process [6]. A high index of clinical suspicion of fungal infections (phaeohyphomycosis) should always be considered in immunocompromised patients, specifically in patients with a history of diabetics, old age, travel to tropics for early treatment [4]. We report a rare case of *Phaeoacremonium* spp. incidentally detected in FNAC and later confirmed by fungal culture.

## Case presentation

**Fig. 1:**
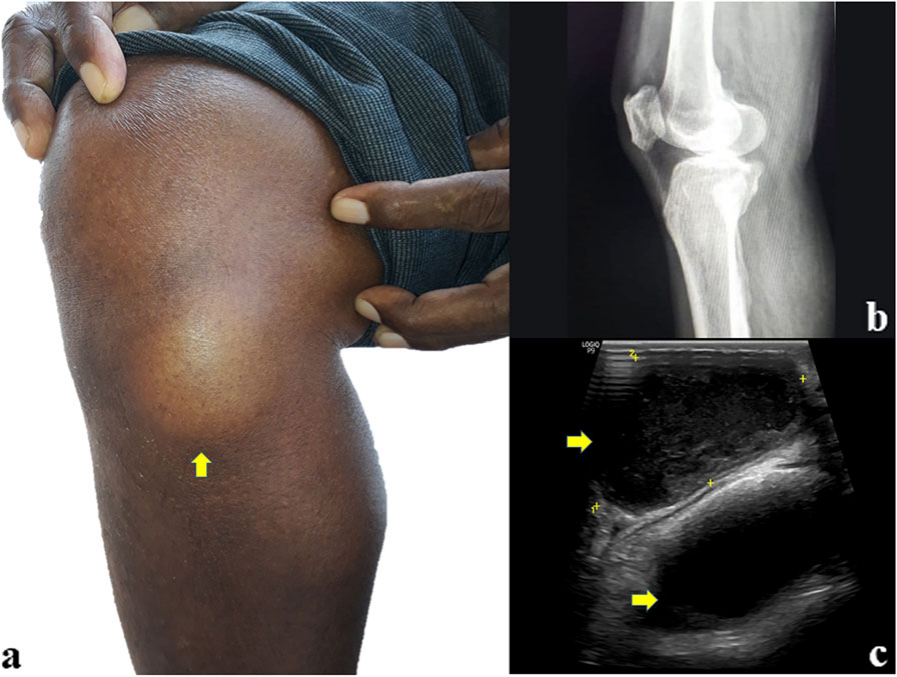
**a** Clinical picture of patient showed a nodule in subcutaneous location below right knee joint in anteromedial aspect with firm consistency and measuring 4x3cm approximately. **b** X-ray revealed no bony involvement; **c** USG of medial aspect of right knee showed two well defined oval anechoic thick-walled lesions measuring 4.5 × 2.6 cm and 4.0 × 2.4 cm in the subcutaneous plane

A 67-year-old male daily construction labourer by profession, presented to surgical outpatient department with a subcutaneous nodule in the medial aspect of the right knee joint for 10 years. He had no significant history of any trauma. On examination the nodule was in a subcutaneous location with firm consistency, slightly tender, restricted mobility, not fixed to underlying structures and was measuring 4x3cm (Fig. 1a).

Laboratory investigations revealed random blood sugar - 452 mg/dL, HbA1c value was 13% (estimated average glucose / eAG – 326 mg/dL) (Normal HbA1c < 5.7%). His hemogram, liver function and renal function tests was within normal limits. Rapid lateral flow assay tests for Human immunodeficiency virus, Hepatitis B, and Hepatitis C were negative. Plain X-ray of knee joint revealed no bony lesion (Fig. 1b). Ultrasonography of medial aspect of right knee showed two well defined oval anechoic thick-walled lesions measuring 4.5 × 2.6 cm and 4.0 × 2.4 cm in the subcutaneous plane, below knee joint in anteromedial aspect. Colour Doppler shows no significant vascular uptake (Fig. 1c). A clinical suspicion of lipoma was made and sent for FNAC.

**Fig. 2:**
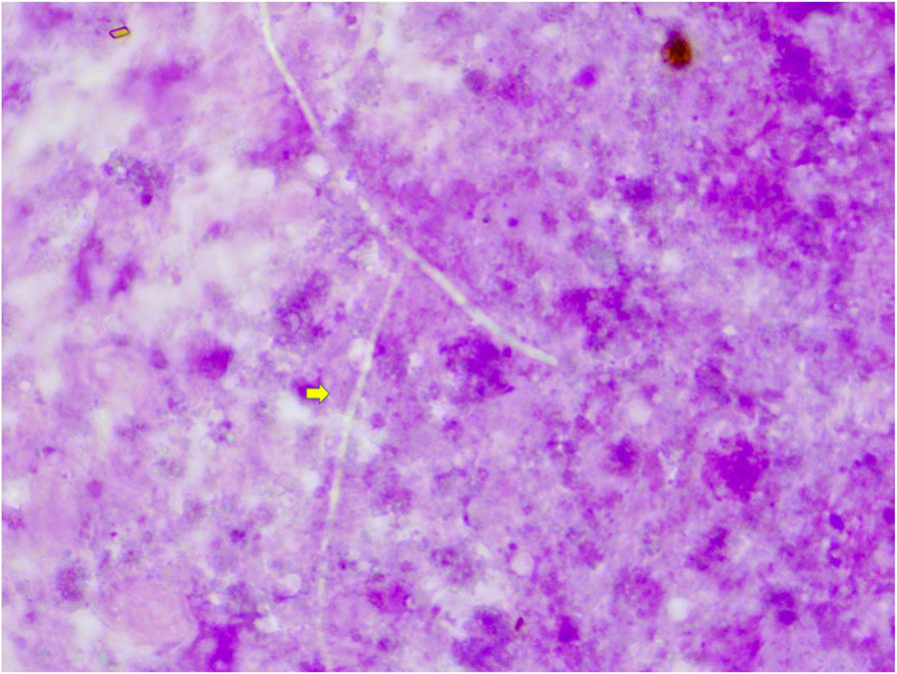
Rapid onsite staining showing the presence of negatively stained acute angle branched hyphae *(arrow)* along with inflammatory background. (Tol Blue, × 100)

FNAC with Rapid On-Site Evaluation (ROSE) was done using toluidine blue using a 22-gauge needle and 5 ml syringe. Aspirate of 2 ml pus-like material. ROSE of slide revealed the presence of negatively stained acute angle branched hyphae along with inflammatory background (Fig. 2). The slides were sent for routine stains.

**Fig. 3:**
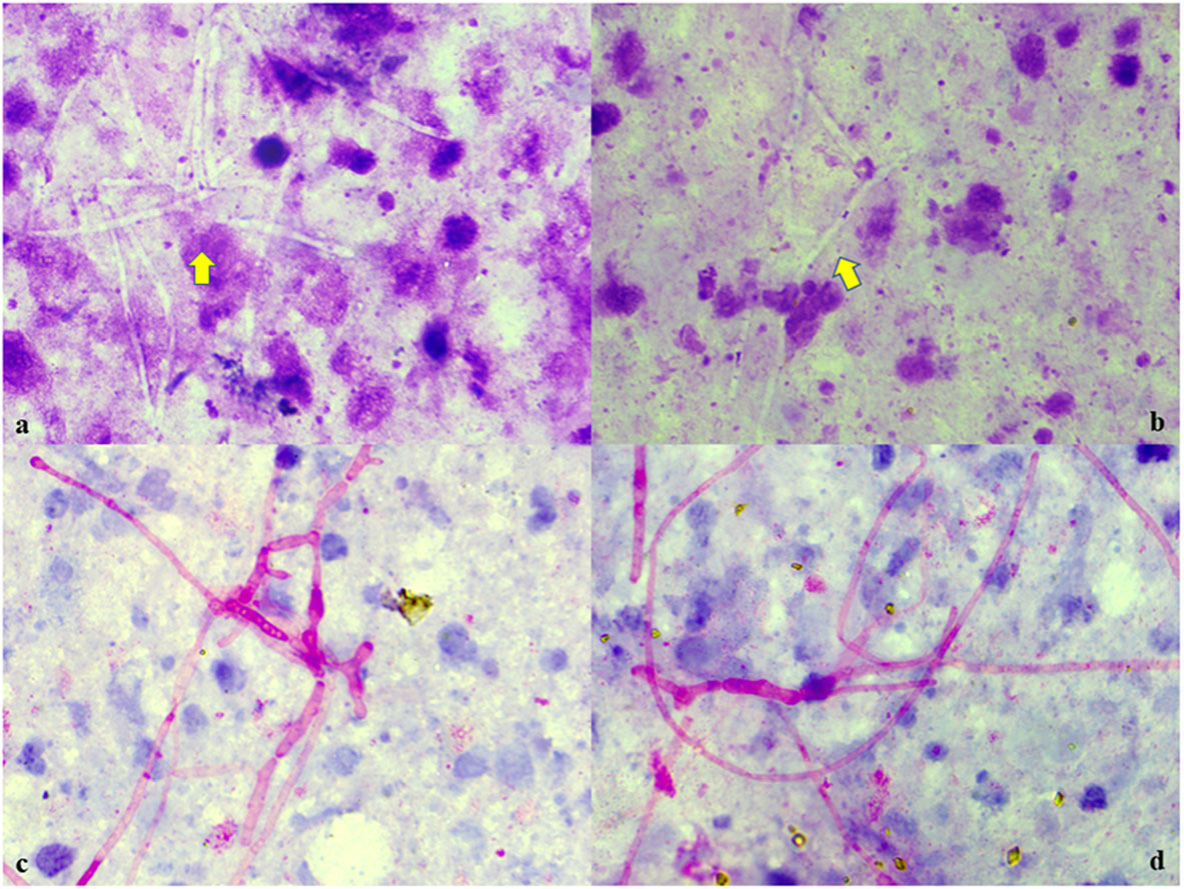
**a**, **b** Cytosmears showing numerous fungal septate hyphae with acute angle branching along with plenty of degenerated inflammatory cells, histiocytes, nuclear debris, Splendore-Hoeppli phenomenon. (Geimsa, × 100); **c**, **d** Periodic acid-Schiff (PAS) stain was positive for the fungal septate branching hyphae. (PAS, × 100)

**Fig. 4:**
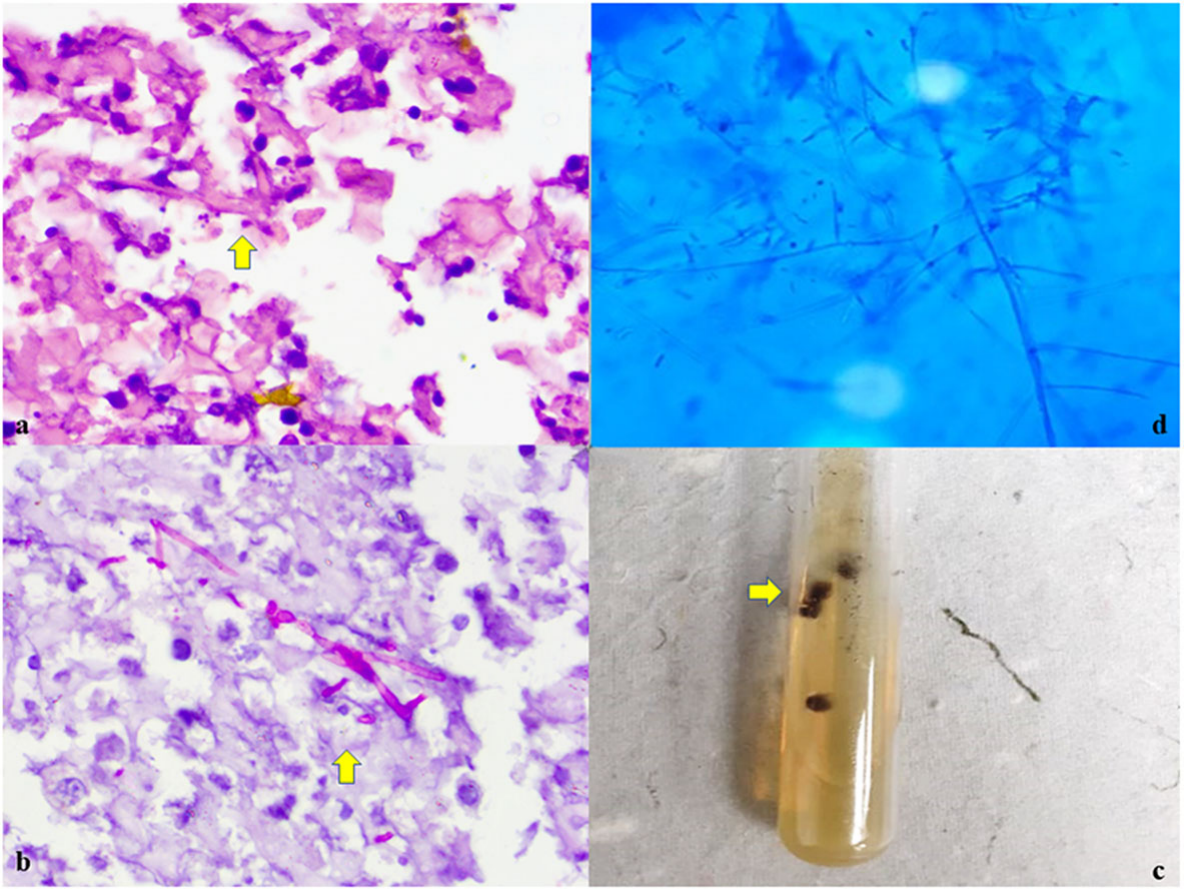
**a**, **b** Microscopy showing numerous fungal hyphae with acute angle branching and inflammatory cells with PAS positivity. (H&E, × 100; PAS, × 100); **c** SDA slant showing surface greyish beige to black color and developed clusters of aerial hyphae; **d** Lactophenol cotton blue (LPCB) mount showing young hyphae forming thick bundles (fascicles), cylindrical phialides along the hyphae and oblong conidia. (LPCB, × 40)

Cytosmears showed plenty of inflammatory cells comprising of neutrophils, lymphocytes, histocytes, nuclear debris, and eosinophilic bodies / Splendore-Hoeppli phenomenon along with numerous fungal septate hyphae with acute angle branching (Fig. 3a,b). The fungal hyphae were positive for Periodic acid-Schiff (PAS) stain (Fig. 3c,d). Gram stain and Ziehl-Neelsen stain were negative. Cell block sample also revealed similar fungal branching hyphae with PAS positivity (Fig. 4a, b).

A diagnosis of inflammatory lesion possibly due to fungal aetiology was given on FNAC & Cell block. The aspirated sample was sent for fungal culture. The fungal culture was done only on Sabouraud’s dextrose agar (SDA) slant and incubated at 37 °C / 25 °C. Growth on tube was noted on 3rd week of incubation. SDA slant showed surface greyish beige to black color and developed clusters of aerial hyphae (Fig. 4c).

Microscopic findings on Lactophenol cotton blue (LPCB) mount showed young hyphae with brownish appearance and side by side to form thick bundles (fascicles), cylindrical phialides along the hyphae and oblong conidia were present gathering in clusters at end of phialide (Fig. 4d). Hence a diagnosis of *Phaeoacremonium parasiticum* was given.

The patient was advised oral itraconazole 100 mg BD for 1 month and currently under follow up with subsidence of the lesion.

## Discussion & Conclusion

Phaeohyphomycosis an opportunistic mycotic infection caused by an heterogeneous group of phaeoid pigmented fungi (Black mold) found widely disseminated in nature [4, 7]. Morphologically these dematiaceous fungi have melanin like pigment and are septate hyphae with pseudohyphae, sometimes co-existing with yeast forms [4]. The morphology of *Phaeoacremonium* genus is intermediate between *Acremonium* and *Phialophora* genus and all are pathogens implicated in opportunistic infections [3].

Crous et al. in 1996 had proposed a species of *Phaeoacremonium parasiticum* with distinctive microscopic features [8]. In humans infected with *Phaeoacremonium,* cutaneous or subcutaneous lesions [7, 9] is the most common, but there have been reported cases of onychomycosis, endophthalmitis, central nervous system involvement, endocarditis, and disseminated disease (fungemia) [4, 9]. *Phaeoacremonium* species reported to cause human infection have included *P. parasiticum, P. rubrigenum, P. alvesii, P. krajdenii, P. amstelodamense, P. griseorubrum, P. tardicrescens*, and *P. venezuelense.* Most are reported to be associated with solid organ transplants [10]. Although there are reports of the fungus in various microbiology journals, however there are very few reports of cytological detection in literature [4].

The relative rarity of this fungus has led to difficulty in the identification of *Phaeoacremonium* species in cultural and microscopic fields [1]. Although difficult, fungal and microscopic features can be used for the identification of the *Phaeoacremonium* species as flat predominantly felty colonies with a woolen texture. These colonies are brown in color with varying shades of pale to dark brown. The mycelium has septate hyphae either singly or in bundles of fascicles. The classic morphologic characters are the combination of conidiophore, phialides, and allantoid conidia.

Molecular tools may be used based on the analysis of the β-tubulin gene sequencing by PCR, is available in limited centers [11]. Patients are usually rural workers with daily contact with soil, wood splinters, thorns, or other trauma-causing objects [7], so is our patient who is a daily laborer.

Fine needle aspiration is a basic and essential technique that is minimally invasive and used to investigate superficial or deep-seated lesions. Rapid on-site evaluation (ROSE) is an add on to the routine FNAC in providing a provisional diagnosis and collection of extra samples for cell block, special stains, fungal cultures, and molecular studies [6, 12]. In our case also ROSE using 1% aq. toluidine blue had helped in arriving at the diagnosis.

In the absence of granuloma, giant cells, negative ZN stain we were able to exclude tuberculosis, sarcoidosis, giant cell tumor. Fungal morphology was helpful to exclude the possibility of Rhinosporidiosis & Aspergillosis [4, 5]. The other differential diagnosis can be chromoblastomycosis which are more superficial disease with sclerotic bodies, micro abscesses, and can be surgically managed [13].

There is no standardized treatment for these rare fungal infections. However, surgical wound excision can be combined with systemic Itraconazole therapy for several months [14]. Antifungals such as amphotericin, fluconazole, posaconazole, ketoconazole, terbinafine, and 5-fluorocytosine (5FC) [15, 16] may be for patients with severe disease, poor response or hepatic toxicity to itraconazole [4].

*P. parasiticum* is an emerging opportunistic fungal infection with limited knowledge regarding the clinical presentation, treatment protocol. A high index of suspicion with confirmation by cytological, histopathological, special stains, culture and molecular techniques for early appropriate treatment is required.

## Data Availability

All the data regarding the findings are available within the manuscript.

## Abbreviations

eAG: Estimated average glucose
FNAC: Fine needle aspiration cytology
LPCB: Lactophenol cotton blue
ROSE: Rapid on-site evaluation
PAS: Periodic acid Schiff
SDA: Sabouraud’s dextrose agar
ZN: Ziehl-Neelsen
5FC: 5-fluorocytosine
